# Industry-supported meta-analyses compared with meta-analyses with non-profit or no support: Differences in methodological quality and conclusions

**DOI:** 10.1186/1471-2288-8-60

**Published:** 2008-09-09

**Authors:** Anders W Jørgensen, Katja L Maric, Britta Tendal, Annesofie Faurschou, Peter C Gøtzsche

**Affiliations:** 1 The Nordic Cochrane Centre, Department 3343, Rigshospitalet, Blegdamsvej 9, DK-2100 Copenhagen, Denmark

## Abstract

**Background:**

Studies have shown that industry-sponsored meta-analyses of drugs lack scientific rigour and have biased conclusions. However, these studies have been restricted to certain medical specialities. We compared all industry-supported meta-analyses of drug-drug comparisons with those without industry support.

**Methods:**

We searched PubMed for all meta-analyses that compared different drugs or classes of drugs published in 2004. Two authors assessed the meta-analyses and independently extracted data. We used a validated scale for judging the methodological quality and a binary scale for judging conclusions. We divided the meta-analyses according to the type of support in 3 categories: industry-supported, non-profit support or no support, and undeclared support.

**Results:**

We included 39 meta-analyses. Ten had industry support, 18 non-profit or no support, and 11 undeclared support. On a 0–7 scale, the median quality score was 6 for meta-analyses with non-profit or no support and 2.5 for the industry-supported meta-analyses (P < 0.01). Compared with industry-supported meta-analyses, more meta-analyses with non-profit or no support avoided bias in the selection of studies (P = 0.01), more often stated the search methods used to find studies (P = 0.02), searched comprehensively (P < 0.01), reported criteria for assessing the validity of the studies (P = 0.02), used appropriate criteria (P = 0.04), described methods of allocation concealment (P = 0.05), described methods of blinding (P = 0.05), and described excluded patients (P = 0.08) and studies (P = 0.15). Forty percent of the industry-supported meta-analyses recommended the experimental drug without reservations, compared with 22% of the meta-analyses with non-profit or no support (P = 0.57).

In a sensitivity analysis, we contacted the authors of the meta-analyses with undeclared support. Eight who replied that they had not received industry funding were added to those with non-profit or no support, and 3 who did not reply were added to those with industry support. This analysis did not change the results much.

**Conclusion:**

Transparency is essential for readers to make their own judgment about medical interventions guided by the results of meta-analyses. We found that industry-supported meta-analyses are less transparent than meta-analyses with non-profit support or no support.

## Background

The primary source of knowledge used by physicians is the medical literature [1]. The amount of information on health care interventions is hardly manageable, however, and the need for systematic reviews and meta-analyses to aggregate the information and give a basis for rational decision-making is therefore obvious [2]. Systematic reviews and meta-analyses of randomised controlled trials are considered to be the highest level of evidence [3].

Bias in industry-sponsored drug trials is common and the sponsor's product is often favoured [4-7]. Concerning systematic reviews and meta-analyses, little information has been available on sponsor bias until recently. We found that, although the results were similar, industry-supported meta-analyses lacked scientific rigour and were more likely to recommend the experimental drug, compared to Cochrane reviews of the same disease and drugs [8]. A study on anti-hypertensive drugs found that meta-analyses with financial ties to only one drug company were associated with conclusions in favour of that company [9].

For this report, we assessed all meta-analyses indexed on PubMed of drug-drug comparisons published in 2004. We hypothesized that meta-analyses supported by the pharmaceutical industry are of poorer methodological quality and have conclusions favouring the experimental drug, compared to meta-analyses with non-profit or no support.

## Methods

We collected meta-analyses that compared different drugs or classes of drugs, were published in full and in English. A meta-analysis was defined as a review with quantitatively combined data from at least two studies. Meta-analyses that we had authored ourselves and meta-analyses of placebo controlled trials were excluded.

### Literature search

We searched PubMed for meta-analyses published in 2004 in English. We used a validated search strategy [10] and combined it with "Drug Therapy" [MeSH] OR "drug therapy" [Subheading] OR "Pharmaceutical Preparations" [MeSH] OR "drugs".

One author (KLM) reviewed the titles and abstracts of all potentially eligible meta-analyses, and if in doubt, the full text of the article was retrieved. Another author (AWJ) assessed all the included and 10% of the excluded meta-analyses for eligibility. Disagreements were resolved by discussion.

### Data extraction

Two authors independently assessed the included meta-analyses. Data extraction was done unblinded. A third author resolved disagreements.

We used a pilot tested data sheet and extracted data on drugs and diseases; types of support; sources used to identify trials for the review; searches for unpublished trials; and descriptions of concealment of allocation, details of blinding, and excluded patients and trials.

We assessed the methodological quality of the reviews with the Oxman and Guyatt index, which is a validated tool with nine items considering the potential for bias and an overall assessment on a 0–7 scale [11-13]. Furthermore, we judged the review authors' conclusions by assessing whether the experimental intervention was recommended without reservations, or whether it was not recommended (or recommended with reservations) [6].

### Data analysis

We divided the meta-analyses according to the type of support in 3 categories: industry-supported, non-profit support or no support, and undeclared support.

Industry support was defined as authorship, provision of grants to authors of the meta-analysis, or other major assistance such as help with the statistical analysis. We did not consider provision of references or unpublished trial reports as support.

We compared industry-supported meta-analyses with meta-analyses with non-profit support or no support with the Mann-Whitney test for categorical data and with Fisher's exact test for binary data; P values are two-sided.

## Results

**Figure 1:**
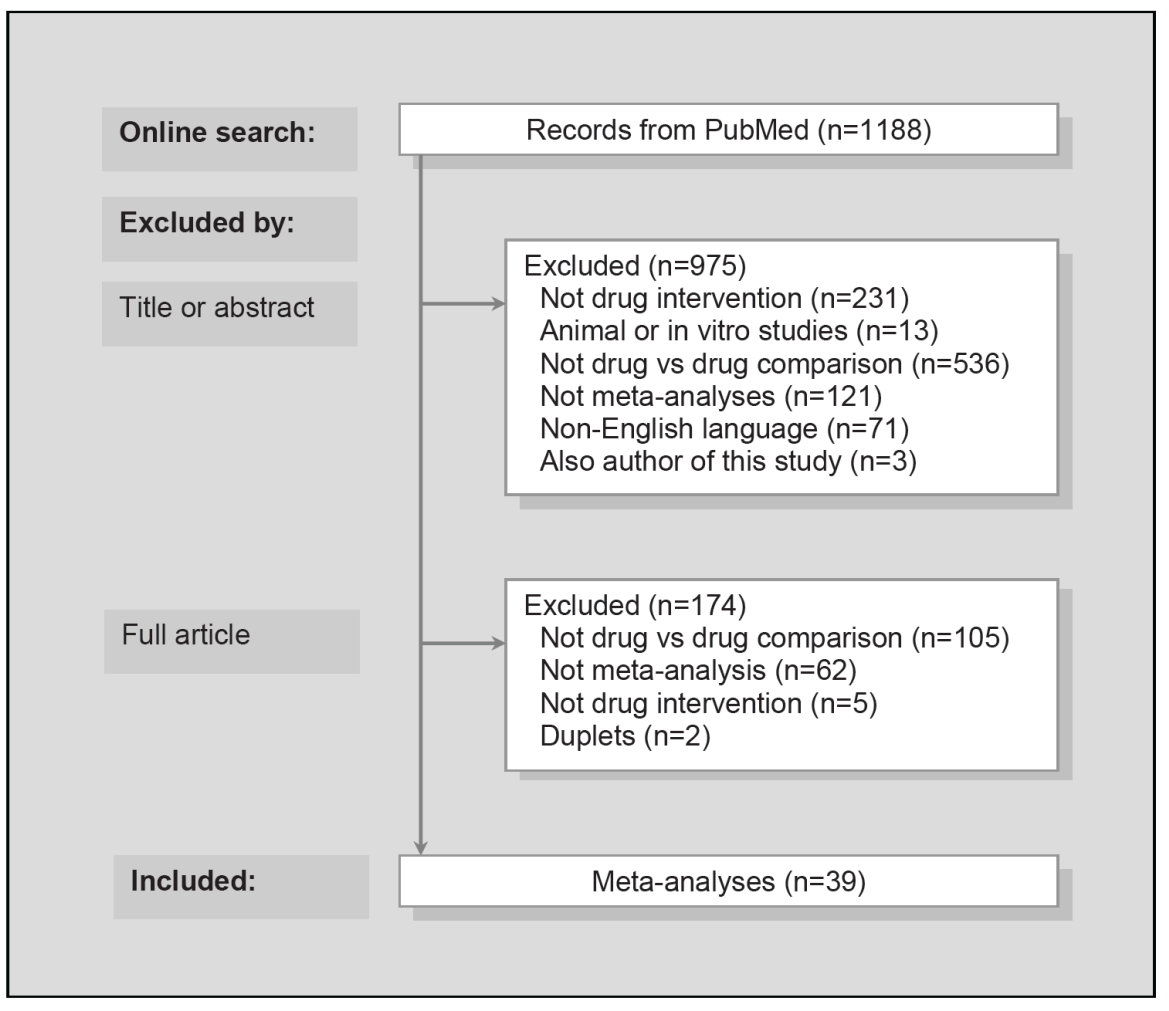
Search for meta-analyses and reasons for exclusion.

The search in PubMed identified 1188 records of meta-analyses. Most were ineligible because they did not compare drugs (Figure 1). We included 39 meta-analyses, 10 of which were Cochrane reviews. Ten had industry support, 18 non-profit or no support, and 11 undeclared support (Table 1 and additional file 1: References of included meta-analyses).

**Table 1:** Characteristics of included meta-analyses.

		**Search strategy incl**.		**Detailed description of**					
					
	**Ref #**	**Medline and CLIB**	**Unpublished studies**	**Allocation concealment**	**Blinding**	**Excluded patients**	**Excluded studies**	**Recommends the experimental drug without reservations**	**Quality score**
**Industry support**	1	✔	✔				✔		4
	2		✔					✔	2
	3						✔		2
	4		✔			✔	✔		3
	5	✔	✔				✔		4
	6							✔	2
	7							✔	1
	8	✔						✔	2
	9	✔					✔		3
	10	✔							6

**Non-profit or no support**	11							✔	2
	12		✔			✔	✔		3
	13	✔	✔				✔	✔	3
	14	✔	✔			✔	✔		6
	15		✔					✔	3
	16	✔	✔				✔		4
	17	✔	✔			✔	✔		7
	18	✔			✔				5
	19	✔	✔				✔	✔	6
	20	✔	✔				✔		3
	21		✔	✔	✔	✔	✔		7
	22	✔	✔	✔	✔	✔	✔		7
	23	✔	✔	✔	✔	✔	✔		7
	24	✔	✔	✔	✔		✔		6
	25	✔	✔	✔		✔	✔		7
	26	✔	✔	✔	✔	✔	✔		7
	27	✔	✔	✔	✔	✔	✔		6
	28	✔	✔				✔		6

**Support not declared**	29	✔				✔			5
	30								3
	31								2
	32	✔	✔		✔	✔	✔		5
	33		✔				✔		3
	34	✔					✔		3
	35						✔		2
	36								1
	37	✔							3
	38		✔			✔	✔	✔	3
	39	✔	✔						2

CLIB: The Cochrane Library

### Methodological quality

The median quality score was 6 for the 18 meta-analyses with non-profit or no support and 2.5 for the ten industry-supported meta-analyses (P < 0.01) (Table 2). More meta-analyses with non-profit or no support avoided bias in the selection of studies (P = 0.01); only five industry-supported meta-analyses reported specific inclusion criteria and two only included studies provided by the supporting company. Meta-analyses with non-profit or no support more often stated the search methods used to find studies (P = 0.02) and searched comprehensively (P < 0.01). Only four (40%) industry-supported meta-analyses searched for unpublished studies and five (50%) searched in the Cochrane Library and MEDLINE. For meta-analyses with non-profit or no support the numbers were 16 (89%) and 14 (78%) respectively. Meta-analyses with non-profit or no support more often reported criteria for assessing the validity of the studies (P = 0.02), used appropriate criteria (P = 0.04), described methods of allocation concealment (P = 0.05), described methods of blinding (P = 0.05), and described excluded patients (P = 0.08) and studies (P = 0.15).

**Table 2:** Methodological quality of meta-analyses.

**Questions**	**Supported by industry**	**Non-profit support or no support**	P value*	**Undeclared support**
	**(N = 10)**	**(N = 18)**		**(N = 11)**
1. Were the search methods used to find evidence on the primary question stated?	6	18	0.02	6
2. Was the search for evidence reasonably comprehensive?	4	17	<0.01	6
3. Were the criteria used for deciding which studies to include reported?	5	17	0.03	10
4. Was bias in the selection of studies avoided?	1	12	0.01	3
5. Were the criteria used for assessing the validity of the included studies reported?	3	15	0.02	5
6. Was the validity of all studies referred to in the text assessed using appropriate criteria?	1	10	0.04	1
7. Were the methods used to combine the findings of the relevant studies reported?	9	17	1.00	10
8. Were the findings of the relevant studies combined appropriately?	7	16	0.46	8
9. Were the conclusions made by the author(s) supported by the data reported?	9	15	1.00	9
Overall quality (1–7) (median score)	2.5	6	<0.01	3

Number of meta-analyses that obtained an affirmative answer to the questions of the Oxman and Guyatt index; and median overall quality score.

* Supported by industry vs non-profit or no support.

### Authors' conclusion

Four industry-supported meta-analyses (40%) recommended the experimental drug without reservations, compared with four meta-analyses with non-profit or no support (22%) (P = 0.57). The supporting companies of all 4 meta-analyses that were recommending the experimental drug without reservation were also selling the drug, but not the control drug. This only applied to 3 of the 6 industry-supported meta-analyses that did not recommend or only recommended the experimental drug with reservations.

### Post hoc sensitivity analyses

We did two sensitivity analyses. First, we excluded the 10 Cochrane reviews from the analysis, as these reviews had high quality scores (median of 7); nine from the meta-analyses with non-profit or no support and one from those with industry support. This resulted in a median quality score of 3 and 2 respectively (P = 0.06).

Second, we contacted the authors of the 11 meta-analyses with undeclared support. Four declared that they had not received any external funding or other type of support (which we exemplified as help with the statistical analyses), four replied that they had received funding from not-profit organisations and three did not reply. We added the eight who replied to the meta-analyses with non-profit or no support and the three who did not reply to the meta-analyses with industry support. This sensitivity analysis did not change the results much, e.g. the median quality score was 5 for meta-analyses with non-profit or no support and 2 for industry-supported meta-analyses (P < 0.01)

## Discussion

We found that meta-analyses with non-profit or no support are of better methodological quality on average than those with industry support. Lack of allocation concealment and blinding, and high attrition rates in randomised controlled trials may bias results of meta-analyses, but if the authors fail to describe these details, the reader is not able to judge if the meta-analysis is reliable. Most industry-supported meta-analyses failed on these counts; this agrees with results we have published previously [8].

### Cochrane reviews

Cochrane reviews seem to have a better methodological quality on average than other meta-analyses [8,14-17].

In this study, ten Cochrane reviews contributed with high methodological quality mainly to meta-analyses with non-profit or no support, as only one Cochrane review was supported by the industry. The policy in the Cochrane Collaboration is that industry support of Cochrane reviews is not allowed [18].

Forty percent of the industry-supported meta-analyses and 22 percent of those with non-profit or no support recommended the experimental drug without reservation. This difference was not statistically significant, but we have previously found a significant difference in a comparison that was closely matched for drugs and diseases (P < 0.01) [8]. A large survey of 124 meta-analyses of anti-hypertensive drugs found that those with financial ties to only one drug company were significantly associated with conclusions in favour of that company [9].

Studies of trials have found similar results. A survey found that none of 56 trial reports of non-steroidal anti-inflammatory drugs supported by the manufacturer presented results that were unfavourable to the company [19]. Another survey found that trials funded by for profit organizations were more likely to recommend the experimental drug as the drug of choice (odds ratio = 5.3) compared with trials funded by non-profit organisations [6]. And recently a study of randomised trials of head-to-head comparisons of statins with other drugs found that not only the conclusions but also the results were in favour of the sponsor's product [7].

### Limitations

We were not blinded, as the layout of some papers, e.g. Cochrane reviews, is unique and impossible to blind. However, blinding when assessing meta-analyses has little impact [20].

A substantial part of the meta-analyses with non-profit or no support were Cochrane reviews and these are made according to the Cochrane Handbook [21]. Andy Oxman participated in the development of the validated index that was used for evaluating methodological quality and he has also participated in developing the Cochrane Handbook.

Our definition of industry support does not distinguish between different amounts of support, and our judgement of support is based on details reported in the meta-analyses. This can theoretically lead to misclassification of the support, as industry support may range from very little to generous, and details about some types of support may be lacking more often than others. However, the definition is operational and we believe that it includes the most important types of industry support. Lack of details or transparency in meta-analyses may also have led to misjudgement of the methodological quality, and it has been argued that the methodological superiority of Cochrane reviews can be explained by the fact that there are no word limits in the Cochrane Library. However, the methodological quality of Cochrane reviews published in regular journals do not seem to differ from Cochrane reviews published in The Cochrane Library [16,17]. Furthermore, important methodological details should always be made available in journals with a word limit, either in the article itself, or in material on the journal's website.

## Conclusion

Transparency is essential for readers to make their own judgment about medical interventions guided by the results of meta-analyses. We found that industry-supported meta-analyses are less transparent than meta-analyses with non-profit support or no support.

## Competing interests

All authors are affiliated with the Nordic Cochrane Centre. We did not receive any funding or support for this study.

## Authors' contributions

AWJ drafted the manuscript and PCG helped. AWJ and KLM participated in writing the protocol, the literature search, data extraction and statistical analysis. BT, AF and PCG participated in data extraction. All authors reviewed and approved the final manuscript.

## Pre-publication history

The pre-publication history for this paper can be accessed here:



## Supplementary Material

Additional file 1 References of included meta-analyses Click here for file
