# Supplementary material for: Industry-supported meta-analyses compared with meta-analyses with non-profit or no support: Differences in methodological quality and conclusions
Source: BMC Med Res Methodol. 2008 Sep 9;8:60. doi: 10.1186/1471-2288-8-60 (PMC2553412; doi:10.1186/1471-2288-8-60)
Supplement: Additional file 1 — References of included meta-analyses [file 1471-2288-8-60-S1.pdf]

References of included meta-analyses.

1. Perrott DA, Piira T, Goodenough B, Champion GD. Efficacy and safety of acetaminophen vs ibuprofen for treating children's pain or fever: a meta-analysis. Arch Pediatr Adolesc Med 2004;158:521-6.

2. Goldstein JL, Eisen GM, Agrawal N, Stenson WF, Kent JD, Verburg KM. Reduced incidence of upper gastrointestinal ulcer complications with the COX-2 selective inhibitor, valdecoxib. Aliment Pharmacol Ther 2004;20:527-38.

3. Watson DJ, Yu Q, Bolognese JA, Reicin AS, Simon TJ. The upper gastrointestinal safety of rofecoxib vs. NSAIDs: an updated combined analysis. Curr Med Res Opin 2004; 20:1539-48.

4. Lee C, Straus WL, Balshaw R, Barlas S, Vogel S, Schnitzer TJ. A comparison of the efficacy and safety of nonsteroidal antiinflammatory agents versus acetaminophen in the treatment of osteoarthritis: a meta-analysis. Arthritis Rheum 2004;51:746-54.

5. Wegman A, van der Windt D, van Tulder M, Stalman W, de Vries T. Nonsteroidal antiinflammatory drugs or acetaminophen for osteoarthritis of the hip or knee? A systematic review of evidence and guidelines. J Rheumatol 2004;31:344-54.

6. Camma C, Di Bona D, Schepis F, Heathcote EJ, Zeuzem S, Pockros PJ, Marcellin P, Balart L, Alberti A, Craxi A. Effect of peginterferon alfa-2a on liver histology in chronic hepatitis C: a meta-analysis of individual patient data. Hepatology 2004; 39:333-42.

7. Strutt K, Caplan R, Hutchison H, Dane A, Blasetto J. More Western hypercholesterolemic patients achieve Japan Atherosclerosis Society LDL-C goals with rosuvastatin therapy than with atorvastatin, pravastatin, or simvastatin therapy. Circ J 2004;68:107-13.

8. Bohle A, Bock PR. Intravesical bacille Calmette-Guerin versus mitomycin C in superficial bladder cancer: formal meta-analysis of comparative studies on tumor progression. Urology 2004;63:682-6.

9. Moyle G, Sawyer W, Law M, Amin J, Hill A. Changes in hematologic parameters and efficacy of thymidine analogue-based, highly active antiretroviral therapy: a meta-analysis of six prospective, randomized, comparative studies. Clin Ther 2004;26:92-7.

10. Deane KHO, Spieker S, Clarke CE. Catechol-O-methyltransferase inhibitors versus active comparators for levodopa-induced complications in Parkinson's disease. Cochrane Database of Systematic Reviews 2004, Issue 4. Art. No.: CD004553. DOI: 10.1002/14651858.CD004553.pub2.

11. Mayo-Smith MF, Beecher LH, Fischer TL, Gorelick DA, Guillaume JL, Hill A, Jara G, Kasser C, Melbourne J. Management of alcohol withdrawal delirium. An evidence-based practice guideline. Arch Intern Med 2004;164:1405-12.

12. Casey JR, Pichichero ME. Meta-analysis of cephalosporins versus penicillin for treatment of group A streptococcal tonsillopharyngitis in adults. Clin Infect Dis 2004;38:1526-34.

13. Colman I, Brown MD, Innes GD, Grafstein E, Roberts TE, Rowe BH. Parenteral metoclopramide for acute migraine: meta-analysis of randomised controlled trials. BMJ 2004;329:1369-73.

14. Wan S, Quinlan DJ, Agnelli G, Eikelboom JW. Thrombolysis compared with heparin for the initial treatment of pulmonary embolism: a meta-analysis of the randomized controlled trials. Circulation 2004;110:744-9.

15. Barbui C, Guaiana G, Hotopf M. Amitriptyline for inpatients and SSRIs for outpatients with depression? Systematic review and meta-regression analysis. Pharmacopsychiatry 2004;37:93-7.

16. Gisbert JP, Pajares JM. Esomeprazole-based therapy in Helicobacter pylori eradication: a meta-analysis. Dig Liver Dis 2004;36:253-9.

17. Bukirwa H, Garner P, Critchley J. Chlorproguanil-dapsone for treating uncomplicated malaria. Cochrane Database of Systematic Reviews 2004, Issue 4. Art. No.: CD004387. DOI: 10.1002/14651858.CD004387.pub2.

18. Dundar Y, Dodd S, Strobl J, Boland A, Dickson R, Walley T. Comparative efficacy of newer hypnotic drugs for the short-term management of insomnia: a systematic review and meta-analysis. Hum Psychopharmacol 2004;19:305-22.

19. Gisbert JP, Khorrami S, Carballo F, Calvet X, Gené E, Dominguez-Muñoz JE. H. pylori eradication therapy vs. antisecretory non-eradication therapy (with or without long-term maintenance antisecretory therapy) for the prevention of recurrent bleeding from peptic ulcer. Cochrane Database of Systematic Reviews 2004, Issue 2. Art. No.: CD004062. DOI: 10.1002/14651858.CD004062.pub2.

20. Gisbert JP, Khorrami S, Calvet X, Pajares JM. Pantoprazole based therapies in Helicobacter pylori eradication: a systematic review and meta-analysis. Eur J Gastroenterol Hepatol 2004;16:89-99.

21. Adams N, Bestall JM, Lasserson TJ, Jones PW. Inhaled fluticasone versus inhaled beclomethasone or inhaled budesonide for chronic asthma. The Cochrane Database of Systematic Reviews 2004, Issue 2. Art. No.: CD002310. DOI: 10.1002/14651858.CD002310.pub2.

22. Afolabi BB, Okoromah CN. Intramuscular arteether for treating severe malaria. Cochrane Database of Systematic Reviews 2004, Issue 4. Art. No.: CD004391. DOI: 10.1002/14651858.CD004391.pub2.

23. Ducharme FM, Di Salvio F. Anti-leukotriene agents compared to inhaled corticosteroids in the management of recurrent and/or chronic asthma in adults and

children. Cochrane Database of Systematic Reviews 2004, Issue 1. Art. No.: CD002314. DOI: 10.1002/14651858.CD002314.pub2.

24. Prasad K, Singhal T, Jain N, Gupta PK. Third generation cephalosporins versus conventional antibiotics for treating acute bacterial meningitis. Cochrane Database of Systematic Reviews 2004, Issue 2. Art. No.: CD001832. DOI: 10.1002/14651858.CD001832.pub2.

25. Holdgate A, Pollock T. Nonsteroidal anti-inflammatory drugs (NSAIDS) versus opioids for acute renal colic. Cochrane Database of Systematic Reviews 2004, Issue 1. Art. No.: CD004137. DOI: 10.1002/14651858.CD004137.pub3.

26. Gibson RC, Fenton M, da Silva Freire Coutinho E, Campbell C. Zuclopenthixol acetate for acute schizophrenia and similar serious mental illnesses. Cochrane Database of Systematic Reviews 2004, Issue 3. Art. No.: CD000525. DOI: 10.1002/14651858.CD000525.pub2.

27. Shepherd J, Brodin H, Cave C, Waugh N, Price A, Gabbay J. Pegylated interferon alpha-2a and -2b in combination with ribavirin in the treatment of chronic hepatitis C: a systematic review and economic evaluation. Technol Assess 2004;8:iii-iv, 1-125.

28. Siebenhofer A, Plank J, Berghold A, Narath M, Gfrerer R, Pieber TR. Short acting insulin analogues versus regular human insulin in patients with diabetes mellitus. The Cochrane Database of Systematic Reviews 2004, Issue 4. Art. No.: CD003287. DOI: 10.1002/14651858.CD003287.pub3.

29. Wilson, K. and Mottram, P. A comparison of side effects of selective serotonin reuptake inhibitors and tricyclic antidepressants in older depressed patients: a meta-analysis. Int J Geriatr Psychiatry. 2004 Aug; 19(8):754-62.

30. Angeli F, Verdecchia P, Reboldi GP, Gattobigio R, Bentivoglio M, Staessen JA, Porcellati C. Calcium channel blockade to prevent stroke in hypertension: a meta-analysis of 13 studies with 103,793 subjects. Am J Hypertens 2004;17:817-22.

31. Angeli F, Verdecchia P, Reboldi GP, Gattobigio R, Bentivoglio M, Staessen JA, Porcellati C. Meta-Analysis of effectiveness or lack thereof of angiotensin-converting enzyme inhibitors for prevention of heart failure in patients with systemic hypertension. Am J Cardiol 2004;93:240-3.

32. Quinlan DJ, McQuillan A, Eikelboom JW. Low-molecular-weight heparin compared with intravenous unfractionated heparin for treatment of pulmonary embolism: a meta-analysis of randomized, controlled trials. Ann Intern Med 2004;140:175-83.

33. Hashiguchi M, Ohno K, Nakazawa R, Kishino S, Mochizuki M, Shiga T. Comparison of cilostazol and ticlopidine for one-month effectiveness and safety after elective coronary stenting. Cardiovasc Drugs Ther 2004;18:211-7.

34. Shelley MD, Wilt TJ, Court J, Coles B, Kynaston H, Mason MD. Intravesical bacillus Calmette-Guerin is superior to mitomycin C in reducing tumour recurrence in high-risk superficial bladder cancer: a meta-analysis of randomized trials. BJU Int 2004;93:485-90.

35. Bolon MK, Morlote M, Weber SG, Koplan B, Carmeli Y, Wright SB. Glycopeptides are no more effective than beta-lactam agents for prevention of surgical site infection after cardiac surgery: a meta-analysis. Clin Infect Dis 2004;38:1357-63.

36. Buzdar AU, Vergote I, Sainsbury R. The impact of hormone receptor status on the clinical efficacy of the new-generation aromatase inhibitors: a review of data from first-line metastatic disease trials in postmenopausal women. Breast J 2004;10:211-7.

37. Chen LC, Elliott RA, Ashcroft DM. Systematic review of the analgesic efficacy and tolerability of COX-2 inhibitors in post-operative pain control. J Clin Pharm Ther 2004; 29:215-29.

38. Casey JR, Pichichero ME. Meta-analysis of cephalosporin versus penicillin treatment of group A streptococcal tonsillopharyngitis in children. Pediatrics 2004;113:866-82.

39. Barron ME, Wilkes MM, Navickis RJ. A systematic review of the comparative safety of colloids. Arch Surg 2004;139:552-63.
